# Supplementary material for: Two-step multi-omics modelling of drug sensitivity in cancer cell lines to identify driving mechanisms
Source: PLoS One. 2020 Nov 23;15(11):e0238961. doi: 10.1371/journal.pone.0238961 (PMC7682852; doi:10.1371/journal.pone.0238961)
Supplement: S1 Appendix — Visualizations of the distributions of model performances; drug compounds are sorted according to their target mechanism. (PDF) [file pone.0238961.s001.pdf]

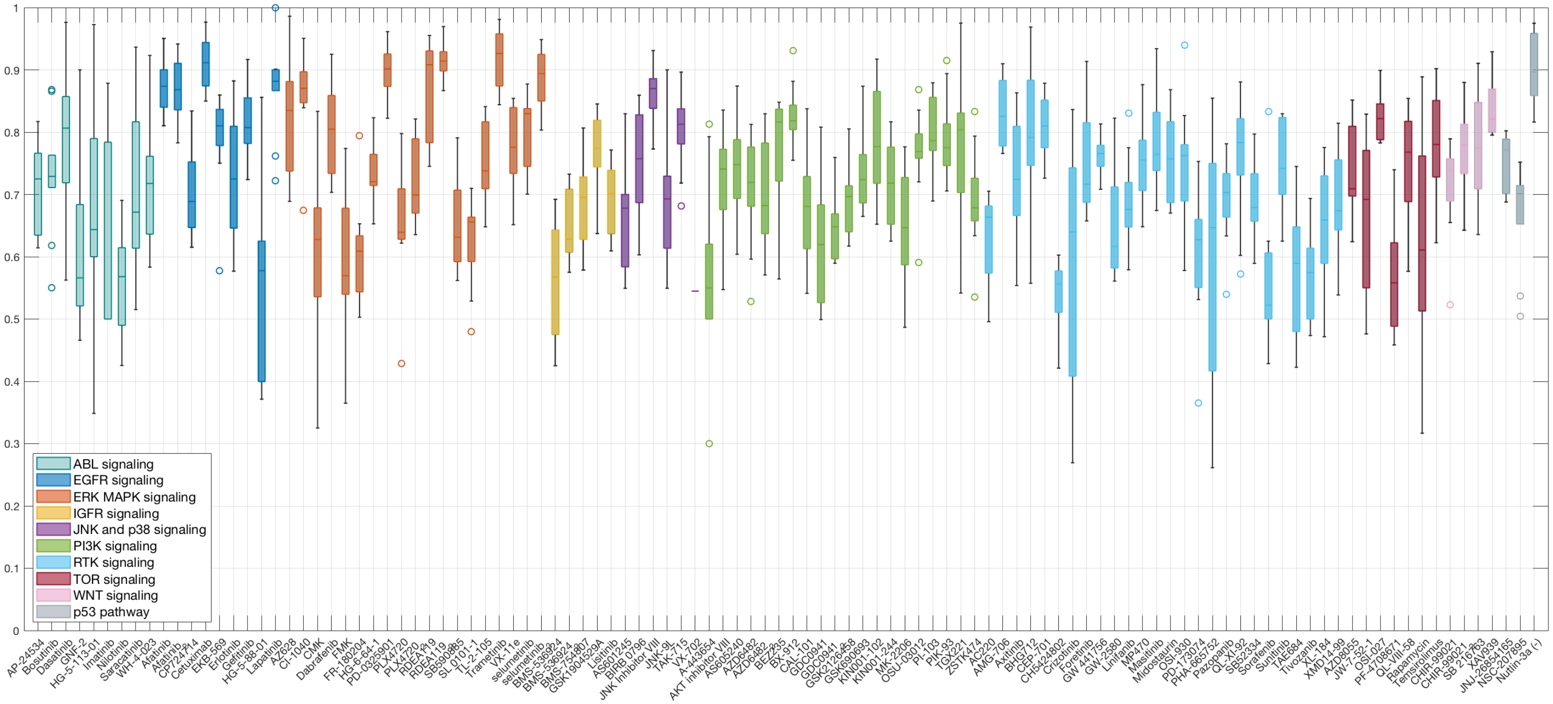

Figure 1: Test ROC-AUCs of the best-performing model for any targeted drug.

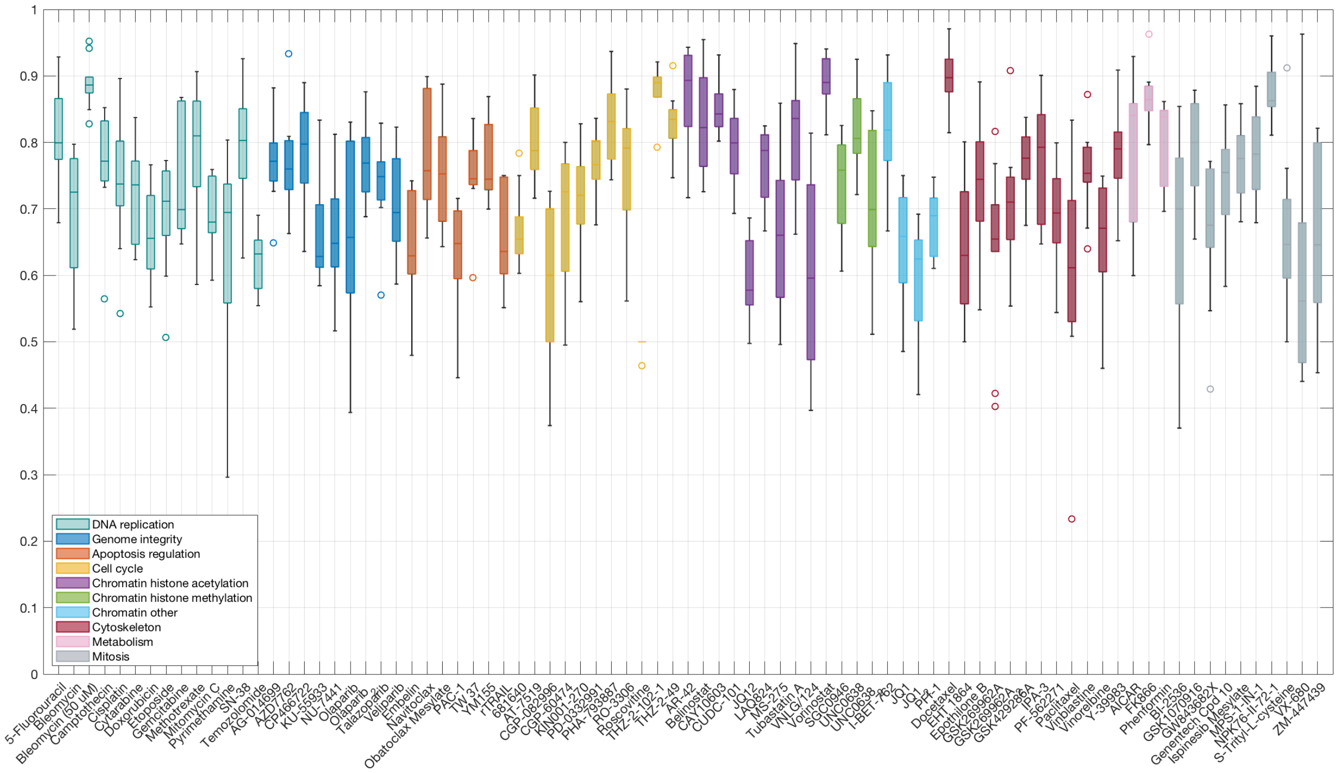

Figure 2: Test ROC-AUCs of the best-performing model for any cytotoxic drug.

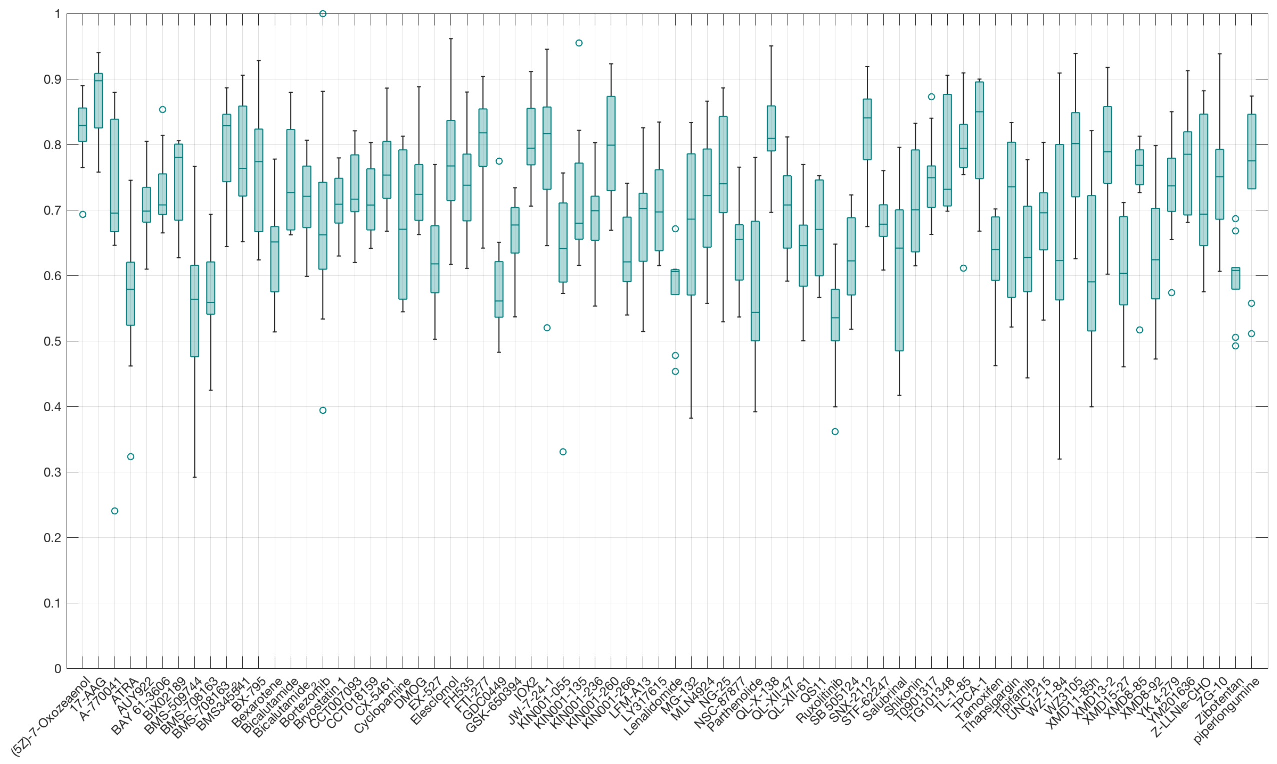

Figure 3: Test ROC-AUCs of the best-performing model for any drug of the class 'other'.
