# Supplementary material for: Two-step multi-omics modelling of drug sensitivity in cancer cell lines to identify driving mechanisms
Source: PLoS One. 2020 Nov 23;15(11):e0238961. doi: 10.1371/journal.pone.0238961 (PMC7682852; doi:10.1371/journal.pone.0238961)
Supplement: S2 Appendix — Heatmaps visualizing the significance of differences between the model performances on distinct drug classes as well as between model performances of models fitted via distinct algorithms. (PDF) [file pone.0238961.s002.pdf]

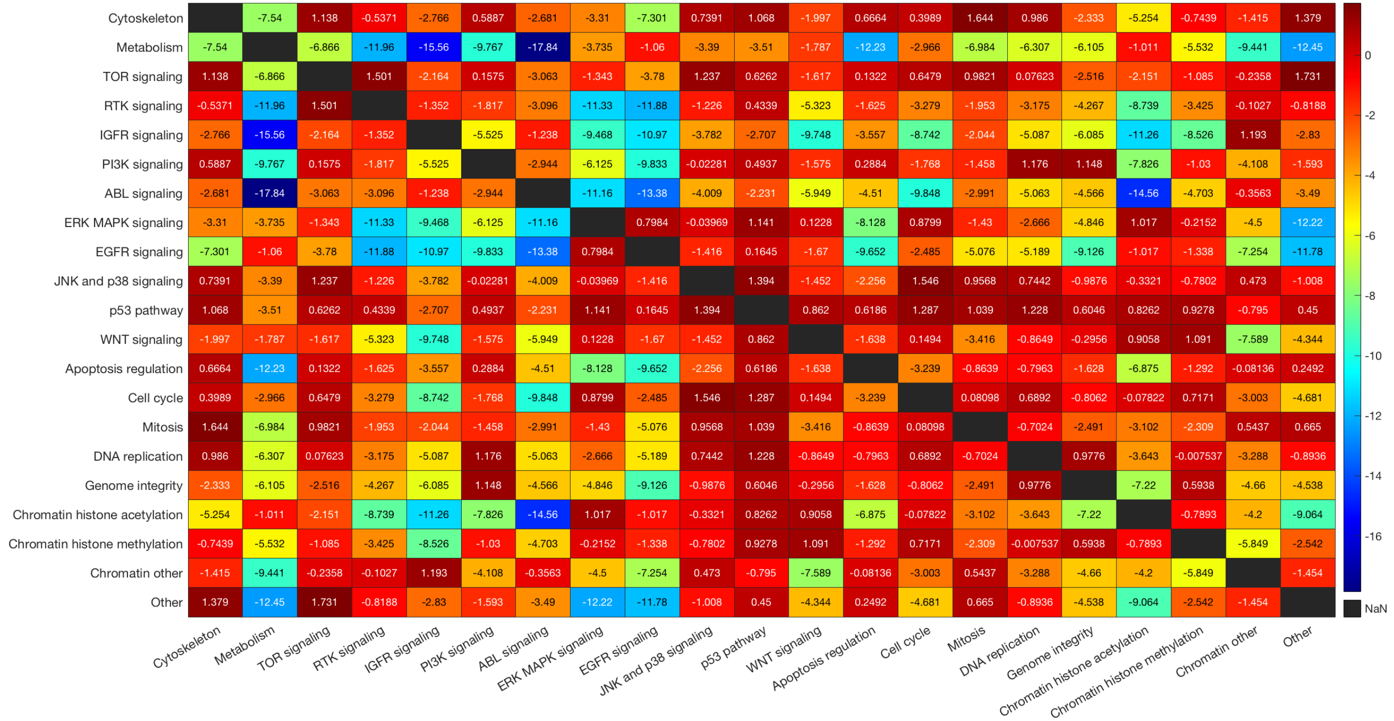

Figure 1: Log_{10}-values of the Bonferroni-corrected p-values obtained by applying a two-sided t-test to any pair of test ROC-AUC distributions of all drug compounds associated with a particular drug class.

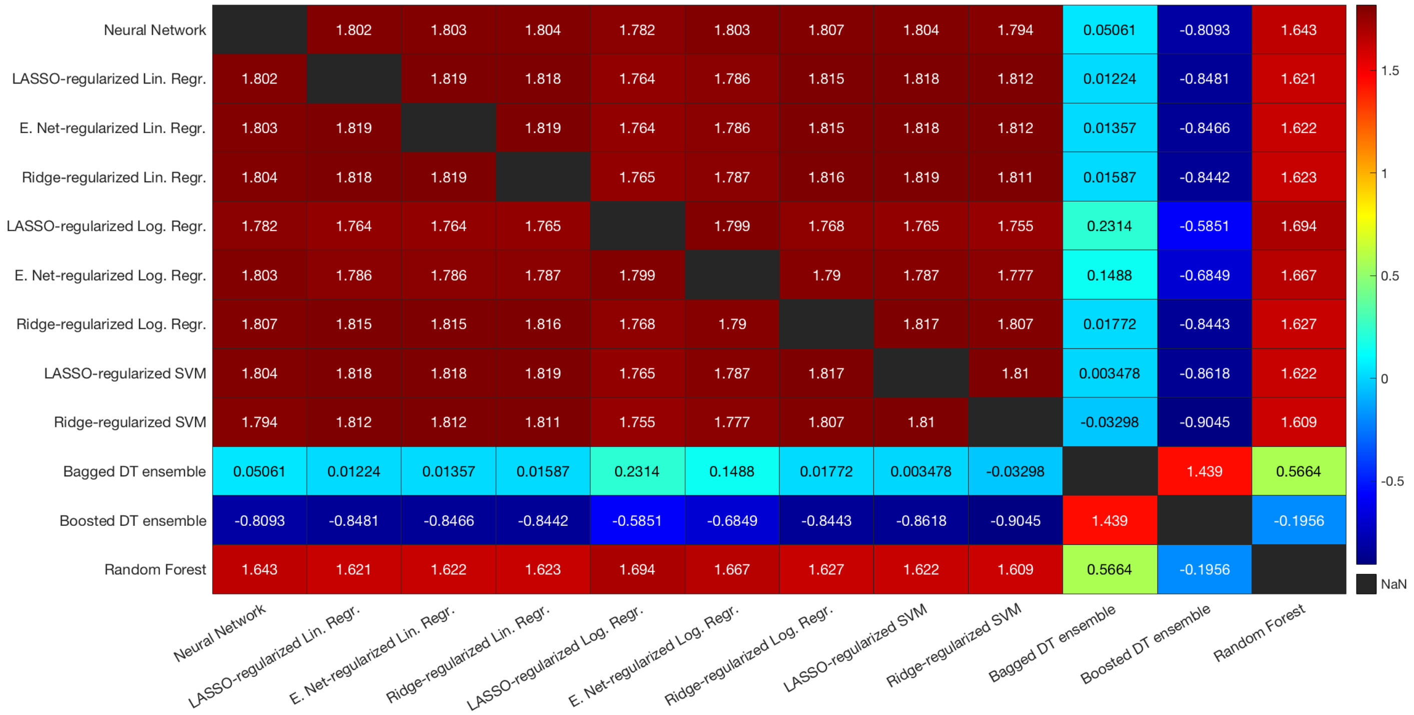

Figure 2: Log_{10}-values of the Bonferroni-corrected p-values obtained by applying a two-sided t-test to any pair of test ROC-AUC distributions of all models caclulated by applying a particular fitting algorithm in the second step.
