# Supplementary material for: Two-step multi-omics modelling of drug sensitivity in cancer cell lines to identify driving mechanisms
Source: PLoS One. 2020 Nov 23;15(11):e0238961. doi: 10.1371/journal.pone.0238961 (PMC7682852; doi:10.1371/journal.pone.0238961)
Supplement: S3 Appendix — Drug class-specific enrichment p-values of drug compounds on which the two-step modelling approach outperforms the one-step models constructed by Jang et al.; overview of drug compounds where one-step models were found to be more predictive and a comparison of overall distributions of performance. (PDF) [file pone.0238961.s003.pdf]

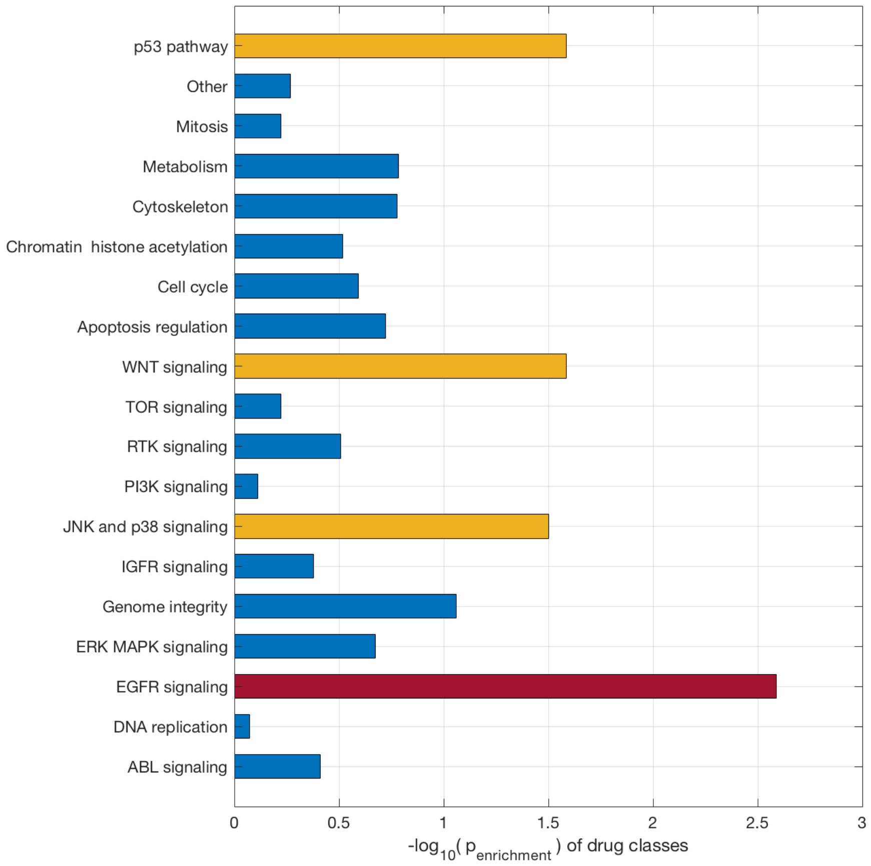

Figure 1: Enrichment of drug classes within the sets of drugs for which at least one model calculated in the two-step modelling workflow outperformed all models calculated by Jang et al. Blue bars correspond to drug classes failing to yield a significant p-value when applying a hypergeometric cumulative distribution function and a significance level of $\alpha = 0.05$; yellow bars indicate drug classes showing significant enrichment before applying the Bonferroni correction for multiple testing, while red bars are associated with classes significantly enriched after correcting for multiple testing. Drug class labels are taken directly from the GDSC annotation.

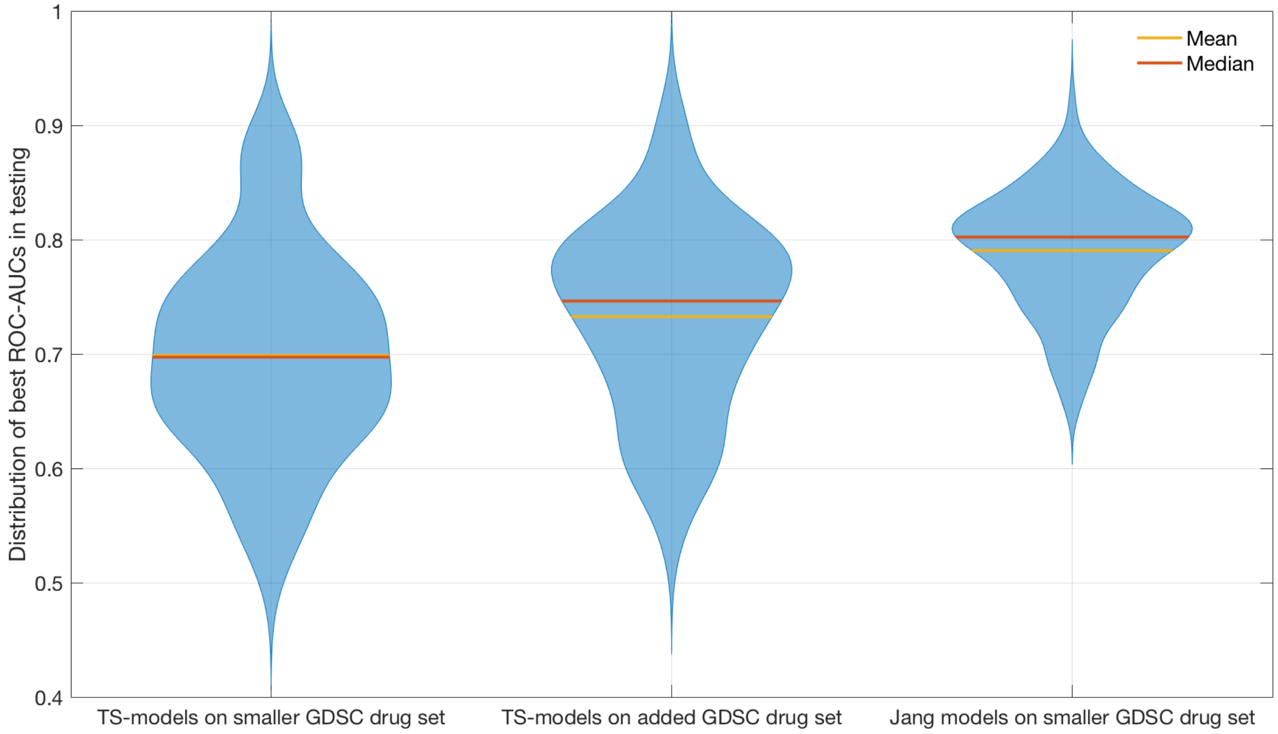

Figure 2: Violin plots of the distribution of concatenated test ROC-AUCs of the most predictive model for any particular drug compound. Models are grouped into three sets: all best-performing models of Jang et al. on the reduced set of drugs featured in a prior version of the GDSC data set (right), all best-performing two-step models calculated on the same set of drugs (left) and all best-performing two-step models constructed on the additional 127 drugs included in the more recent version of the GDSC platform.

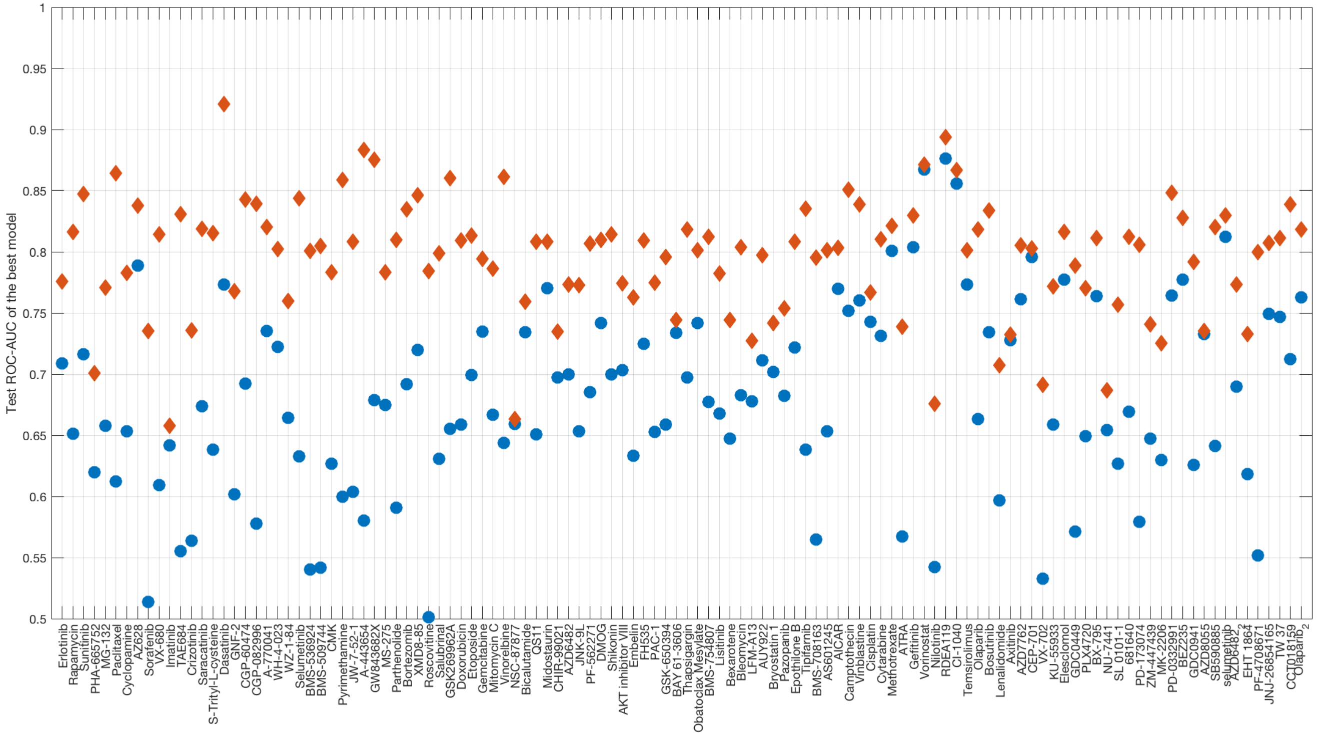

Figure 3: Concatenated test ROC-AUC of the respective best-performing two-step model (blue) for any drug where all two-step model are outperformed by the most predictive straightforward multi-omics model generated in the study of Jang et al. (red).
