# Supplementary material for: Two-step multi-omics modelling of drug sensitivity in cancer cell lines to identify driving mechanisms
Source: PLoS One. 2020 Nov 23;15(11):e0238961. doi: 10.1371/journal.pone.0238961 (PMC7682852; doi:10.1371/journal.pone.0238961)
Supplement: S5 Appendix — List of discrete and continuous features associated with shifts in sensitivity to Docetaxel; visualization of the predictive performances of first-step models and their relative importance, as quantified by second-step algorithms. (PDF) [file pone.0238961.s005.pdf]

Docetaxel

The following table summarizes all discrete features determined by the first-step models to be related to a shift in the responsiveness of the human cancer cell lines featured in the GDSC data base to the drug compound Docetaxel. This includes somatic mutations, copy number variations (CNVs), hypermethylation events and tissue descriptors; these data types are included in the list under the condition that they yield non-constant first-step models in at least five of the ten cross-validation folds.

Each featured data type is associated with a mean relative importance, calculated by using the weights and importance scores fitted by the second-step models, with the exception of the neural network and the naïve Bayes classifier: the absolute values of weights or importance scores assigned by any algorithm to any first-step model are averaged over all folds and then normalized per algorithm in order to yield a measure for an algorithm-specific relative importance for each first-step model. Subsequently, these values are averaged over all algorithms, resulting in a data-type specific mean relative importance score, which is featured in the table below. Values close to one indicate that a first-step model based on one particular data type has been found to contribute most effectively to the prediction. Figure 1 visualizes all calculated algorithm-specific relative importance scores.

The column labelled 'Stability' shows how often a feature is found to be significant over all of the cross-validation folds.

Table 1: Discrete features

Data Type	Mean Relative Importance	Feature	Effect	Stability
CNV	0.09	EGFR	Sensitivity	100%
		CCND1	Sensitivity	100%
		CTTN	Sensitivity	100%
Methylation	0.22	DERL3	Sensitivity	100%

Data Type	Mean Relative Importance	Feature	Effect	Stability
		MYH14	Sensitivity	100%
		TBX18	Sensitivity	100%
		WNK4	Sensitivity	100%
		VPS25	Sensitivity	100%
		KCNC3	Sensitivity	100%
		POU4F1	Sensitivity	100%
		GPR135	Sensitivity	100%
		KCND2	Sensitivity	100%
		ZNF829	Sensitivity	100%
		ZNF568	Sensitivity	100%
		FAM183A	Sensitivity	100%
		ZNF470	Sensitivity	100%
		SVIP	Sensitivity	100%
		HOXA7	Sensitivity	90%
		SLC46A3	Sensitivity	70%
		PAK6	Sensitivity	40%
		C15orf56	Sensitivity	40%
		MT1M	Sensitivity	40%
		SQSTM1	Sensitivity	40%
		MAP1LC3A	Sensitivity	20%
		IL17RE	Sensitivity	20%
		IL17RC	Sensitivity	20%
		TOX2	Resistance	10%
Tissue Type	0.20	Upper Aero-digestive tract	Sensitivity	100%
		Hematopoietic & Lymphoid cells	Resistance	100%

Data Type	Mean Relative Importance	Feature	Effect	Stability
		Central Nervous System	Sensitivity	100%
		Soft Tissue	Sensitivity	80%
		Oesophagus	Sensitivity	60%
		Thyroid	Sensitivity	60%
		Lung	Resistance	10%

The following table holds all statistically significant features identified by fitting the first-step linear regression models on the continuous data types, namely the pathway activation scores and the principal components calculated on basal gene expression data. Mean relative importance and stability values are calculated as described above.

Features are included in the list if their fitted coefficient is associated with a significant p-value in at least six folds, when applying a significance level of $\alpha = 0.05$ and the Bonferroni-correction for multiple testing on eleven pathways and seven principal components, respectively. Mean p-values for such features are calculated over all folds and are included only as approximations.

Table 2: Continuous features

Data Type	Mean Relative Importance	Feature	Mean p-value	Stability
Pathway Activation	0.11	TGFb	10^{-4}	100%
Principal Components	1	PC_1	10^{-10}	100%
		PC_7	10^{-6}	100%
		PC_3	10^{-3}	90%

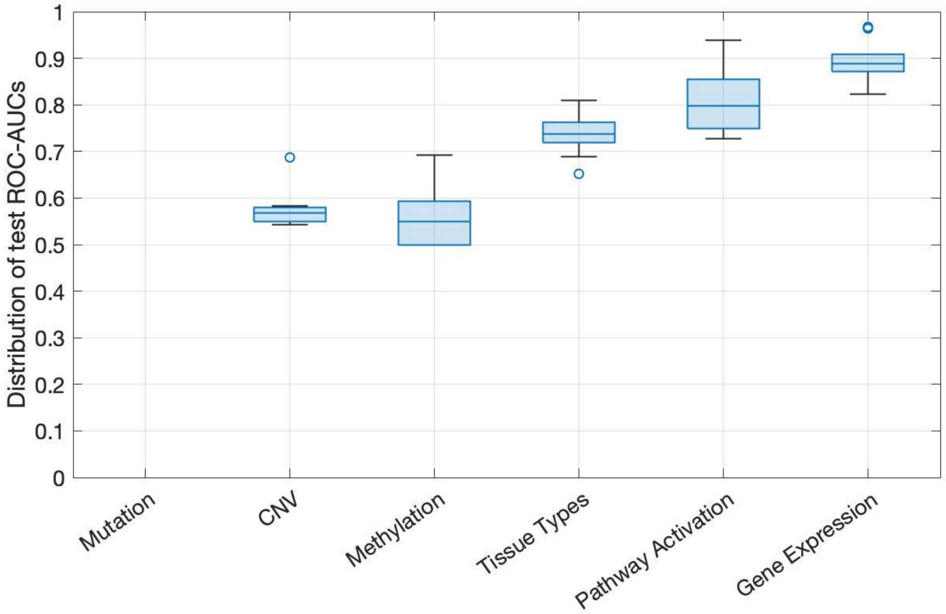

Figure 1: Distributions of the test ROC-AUCs of all non-constant first-step models that are calculated in at least five out of the 10 folds for the drug Docetaxel. Dark blue lines inside the boxes indicate the median values.

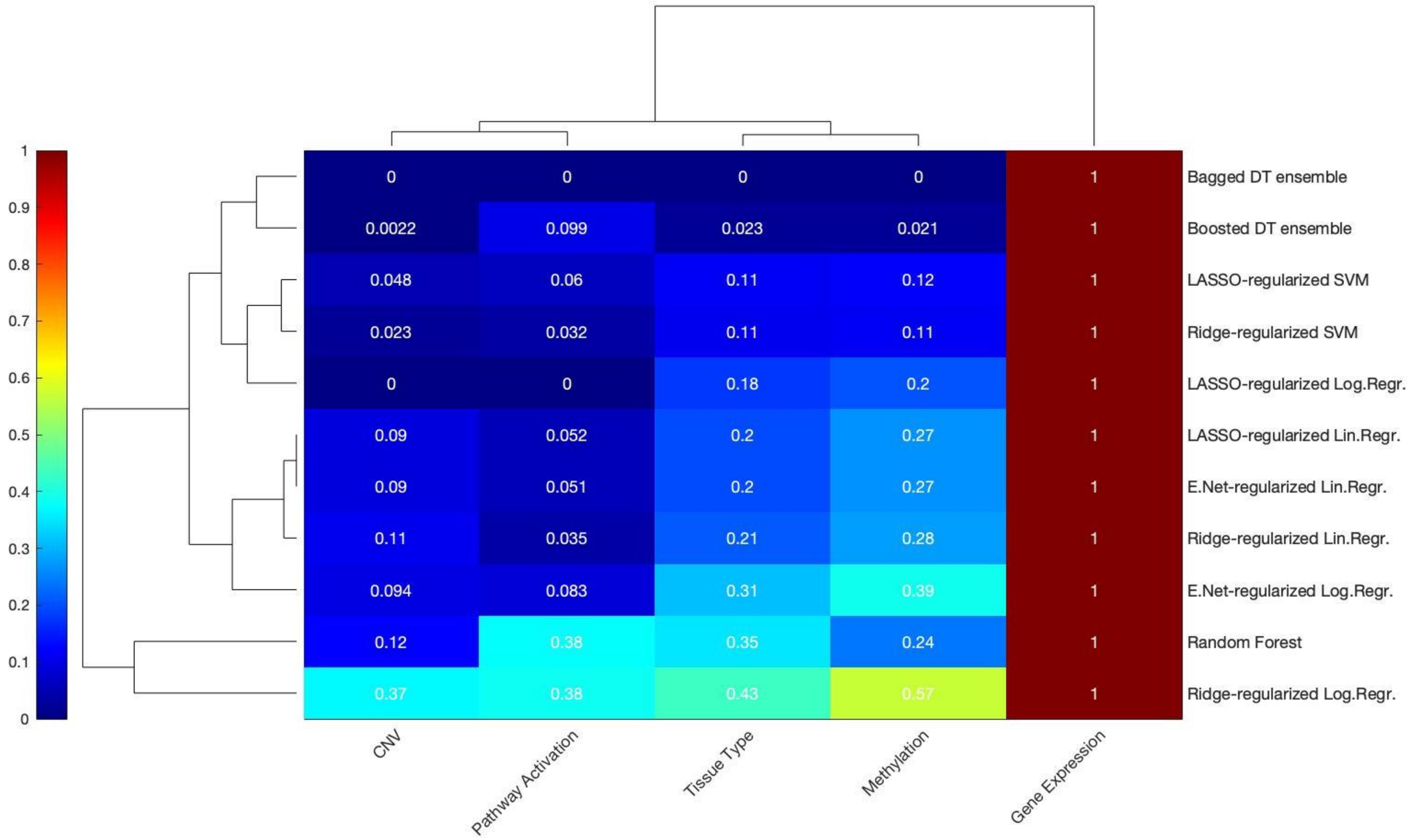

Figure 2: Hierarchically clustered heatmap of the averaged normalized weight and importance scores of all first-step models, indicated here by data type, as determined by the second-step fitting algorithms. Values close to 1 indicate a high importance.
