# Supplementary material for: Two-step multi-omics modelling of drug sensitivity in cancer cell lines to identify driving mechanisms
Source: PLoS One. 2020 Nov 23;15(11):e0238961. doi: 10.1371/journal.pone.0238961 (PMC7682852; doi:10.1371/journal.pone.0238961)
Supplement: S1 Fig — Comprehensive figure of the complete modelling workflow. (PDF) [file pone.0238961.s006.pdf]

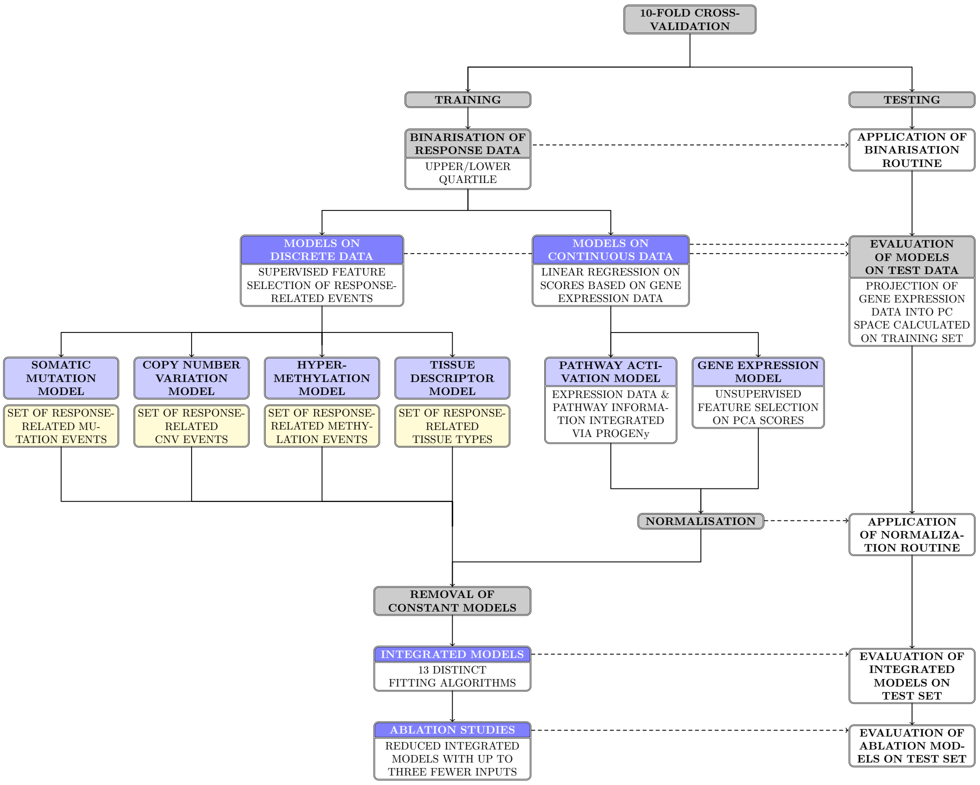

S1 Fig: Comprehensive visualization of the two-step modelling workflow. Boxes colored in light blue correspond to singular models, while boxes colored in medium blue represent sets of models. Yellow boxes are associated with feature sets that hold information about potential discrete drivers of drug responsiveness. Dotted lines represent routines – binarization, normalization or models – that are built using the training data and are subsequently applied to the test data set.
