# Supplementary material for: Two-step multi-omics modelling of drug sensitivity in cancer cell lines to identify driving mechanisms
Source: PLoS One. 2020 Nov 23;15(11):e0238961. doi: 10.1371/journal.pone.0238961 (PMC7682852; doi:10.1371/journal.pone.0238961)
Supplement: S1 Table — Table detailing the settings of model parameters, as they differ from the default settings provided by MATLAB. (PDF) [file pone.0238961.s007.pdf]

S1 Table: Algorithms used in the second step of the two-step models and their respective parameter settings, whenever they differ from the default settings defined by Matlab

Algorithm	Function	Parameter	Parameter Setting
Neural Network	fitnet	Training function	Bayesian Regularization backpropagation, 'trainbr'
		Number of hidden layers	1
		Number of nodes per hidden layer	5
		Train/Test ratio	80/20
Linear regression with LASSO regularization	lasso		
Linear regression with Elastic Net regularization	lasso	Weight of lasso versus ridge optimization	10^{-1}
Linear regression with ridge regularization	lasso	Weight of lasso versus ridge optimization	10^{-3}
Logistic Regression with LASSO regularization	lassoglm	Distribution of response data	binomial
		Number of regularization parameters	100

Algorithm	Function	Parameter	Parameter Setting
		Number of cross validation folds	5
Logistic Regression with Elastic net regularization	lassoglm	Distribution of response data	binomial
		Number of regularization parameters	100
		Number of cross validation folds	5
		Weight of lasso versus ridge optimization	0.5
Logistic Regression with ridge regularization	lassoglm	Distribution of response data	binomial
		Number of regularization parameters	100
		Number of cross validation folds	5
		Weight of lasso versus ridge optimization	10^{-3}
Support Vector Machine with ridge regularization	fitrlinear	Learner	svm
		Regularization	ridge

Algorithm	Function	Parameter	Parameter Setting
Support Vector Machine with LASSO regularization	fitrlinear	Learner	svm
		Regularization	lasso
Naïve Bayes Classifier	fitcnb		
Bagged Decision Tree ensemble	fitrensemble	Method	Bootstrap aggregation for binary regression problems, 'Bag'
		Weak learner	Default decision tree learner template with one decision split
Boosted Decision Tree ensemble	fitrensemble	Weak learner	Default decision tree learner template with one decision split
Random Forest	TreeBagger	Number of trees	50
		Method	Regression
		MinleafSize	20
		OOBPredictorImportance	on
		PredictorSelection	curvature
